# Supplementary material for: Are carbon emissions trading and green financial instruments synergistic? -Comprehensive quantitative research based on content analysis
Source: PLoS One. 2024 Mar 7;19(3):e0298601. doi: 10.1371/journal.pone.0298601 (PMC10919601; doi:10.1371/journal.pone.0298601)
Supplement: S1 Data — (ZIP) [file pone.0298601.s001.zip › Data/地级市Z_F工作报告.zip!/╕ú╜¿/╧├├┼╩╨/└┤╫╘╣1⁄2╓┌║┼í╛┬φ┐╦╩2╛▌═°í┐_2019.docx]

各位代表:

　　现在，我代表市人民政府向大会报告工作，请予审议，并请市政协委员提出意见。

　　一、2018年工作回顾

　　过去一年，习近平总书记亲自向第二十届投洽会等致贺信，对福建、厦门工作作出重要指示批示，给全市人民巨大鼓舞。在以习近平同志为核心的党中央坚强领导下，我们坚持以习近平新时代中国特色社会主义思想为指导，全面贯彻落实党的十九大和十九届二中、三中全会精神，按照省委省政府和市委的决策部署，着力稳增长、促改革、调结构、惠民生、防风险，以“双千亿”工作为抓手，推进高质量发展落实赶超，经济社会保持平稳健康发展。预计全年地区生产总值4791.4亿元，增长7.7%；固定资产投资增长10.1%；财政总收入1283.3亿元，增长8.1%，其中，地方级财政收入754.5亿元，增长8.3%；城乡居民人均可支配收入增幅高于经济增速；居民消费价格上涨1.8%；完成年度节能减排任务。

　　一年来的主要工作和成效:

　　（一）坚持调结构、促转型，发展质量效益取得新成效

　　稳增长措施有力有效。科学研判外部经济形势，有针对性地出台促进有效投资、稳定工业外贸、积极利用外资、促进消费升级、支持民营经济、发展总部经济等一系列政策，密集推出降本减负措施，为企业减负超300亿元。定期举办政银企对接会，完善政策性担保体系，积极缓解企业融资难题。新增“五个一批”项目1242个，总投资9862亿元。规上工业增加值增长8.8%。社会消费品零售总额增长6.6%。获评全国首批服务型制造示范城市。实施“中国制造2025”、促进工业稳增长和转型升级连续两年获国务院表扬。

　　创新步伐明显加快。火炬高新区国家双创示范基地建设获国务院表扬。自创区建设在全省考评中获得第一，累计推出创新事项89项，落地产业科技项目268个。全社会研发投入占GDP比重为3.4%。国家高新技术企业净增200家，累计超1600家，规上高新技术产业增加值占规上工业68%。高新技术成果转化项目增长70%。技术交易合同数及总金额分别占全省58.8%、63.8%。新增国家级企业技术中心4家。3家企业获国家级人工智能创新示范。新增成长型中小企业、小巨人领军企业、技术创新示范企业近千家。新增国际、国家、行业标准189项。每万人拥有有效发明专利数量为全国2.4倍，获评知识产权事业重大进展和突出成就典型城市。国家“千人计划”人才增至110人。累计设立众创空间203家，在孵创业团队4686个。

　　产业链群不断壮大。新增计算机与通讯设备、机械装备、文化创意3条千亿产业链群，累计达8条。331个亿元以上产业项目完成投资340亿元，神州优车、趣店集团、瑞幸咖啡等一批项目签约落地，通富微电子、士兰微电子、宸美三代触控屏等一批项目开工建设，厦钨永磁电机等一批项目增资扩产，ABB工业中心等一批项目建成投产。半导体和集成电路、软件和信息服务、生物医药与健康、新材料等战略性新兴产业分别增长18.6%、17%、20%和18%。旅游业接待游客8900万人次，增长13.7%，举办会议展览9145场，旅游会展业实现总收入1660亿元，增长17.8%。现代物流业总收入1180亿元，增长7.4%，集装箱吞吐量完成1070.2万标箱，保持全球第14位。金融机构本外币存贷款余额分别增长3.7%和8.3%。海洋经济增加值增长10.2%，获批建设国家海洋经济发展示范区。

　　（二）坚持强动力、增活力，改革开放实现新突破

　　重大重点领域改革成效凸显。政府机构改革方案获批实施，机构职能配置进一步优化。“放管服”改革力度加大，取消、下放审批服务事项77项，清理、优化审批特殊环节302项，实施“35证合一”，“一趟不用跑、最多跑一趟”事项占比提升至93%，新版　 “i厦门”实现从网上办到掌上办。全国工程建设项目审批制度改革现场会在我市召开，厦门改革成效得到国务院通报表扬。综合信用指数在36个省会及副省级城市排名第二，营商环境经国家发改委评定居全国试点城市第2位。自贸试验区累计推出361项创新举措，其中全国首创63项，口岸收费降至大陆最低水平，航空维修、融资租赁等16个平台规模不断壮大，获批国家跨境电商综合试验区、集装箱货物过境运输口岸、首批国家文化出口基地。国有企业改革创新取得实效，市属国企资产总额、利润总额分别增长13.2%和13.1%，国贸、建发、象屿进入2018年财富世界500强，国企内部监管、责任追究等机制更加完善。医药卫生体制改革进展顺利，获批“4+7”药品带量采购试点城市，分级诊疗和家庭医生签约服务有序推进。

　　开放步伐持续加快。全年合同利用外资465.9亿元，实际使用外资107.3亿元，分别占全省44.6%和35.2%；进出口总额6002.1亿元，增长3.2%，其中进口增长4%，外贸综合竞争力位居全国第5位。获批外国人144小时过境免签政策。“丝路海运”航线正式开行。接待邮轮96艘次，旅客运输量增长100.8%。荣获国际友城交流合作奖“六连冠”。

　　对台交流合作扎实推进。成功举办第十届海峡论坛、2018年两岸企业家峰会年会、工博会等重大活动。新批台资项目794个，增长15.4%。“小三通”运送旅客174.5万人次，增长8.1%。海运快件增长1.4倍。台湾水果进口量连续11年排名大陆城市第一。国家级两岸青创基地增至7家，在厦台湾创业团队达500个。“厦门惠台60条”及其细则顺利实施，台胞台企“双待遇”得到有效落实。

　　（三）坚持出重拳、破难题，三大攻坚战取得新进展

　　积极防范化解重大风险。完善地方金融风险防控预警平台，建立信贷风险化解工作联席会议制度，健全应急周转机制，有效化解不良贷款。专项整治互联网金融风险，24家网贷机构完成整改验收，309家未营运机构退出市场。清理整顿现金贷、校园贷等，金融市场平稳有序。完善政府债务管理机制，债务风险安全可控。坚决落实稳定房地产市场政策措施，积极推进住房租赁试点工作，加快构建租购并举住房制度，房地产市场健康平稳。

　　加快推进脱贫攻坚。在国家东西部扶贫协作年度考核中位列东部城市首位。各区、镇街全部与临夏州贫困县、乡镇结对。筹集援临帮扶资金4.75亿元，增长188%。国有企业到临夏州投资成立15家公司，开展32个项目合作，实施“临夏特色产品走出去”工程。举办57场专场招聘会，劳务输转到厦就业4429人，带动建档立卡贫困人口就地就近就业3565人。闽宁镇特色产业加快培育。省内扶贫开发稳步推进，援宁援藏援疆援渝等任务有效落实。

　　持续加强污染防治。认真整改中央和省环保督察、国家海洋督察反馈问题，完成23项，其余26项按序时推进。空气质量综合指数在全国169个重点城市排名第七。集中式饮用水源水质达标率保持100%，城市生活污水集中处理率96%。河长制工作成效明显，9条小流域和筼筜湖、五缘湾水环境得到有效治理，小流域周边污染源清退全面完成，国控省控断面水质达标率100%，6处黑臭水体治理通过国家专项督查，近岸海域Ⅰ-Ⅱ类水质面积超过省考核要求。全市土壤环境质量总体良好，危险废物处置利用率96%。完成营造林4.9万亩，改造提升园林绿地532公顷，通过国家森林城市复查。51项生态文明体制改革任务有序推进，率先全国形成生态系统价值核算沿海样本，率先全省开展生态环境损害赔偿试点。

　　（四）坚持拓空间、优环境，城市功能品质得到新提升

　　产城融合加快推进。大力推进闽西南协同发展区建设，首批22个重大项目加快实施。岛外固定资产投资占全市75%，其中新城基地完成投资992.8亿元。集美新城161个项目完成投资202.8亿元，软件园三期营收增长40.3%，机械工业集中区等园区产业集聚发展。环东海域新城334个项目完成投资506亿元，滨海酒店群加快建设，滨海旅游浪漫线一、二期基本建成。马銮湾新城234个项目完成投资243亿元，五纵六横骨干路网基本成形，陆域吹填、生态绿化、公建配套等进展顺利。同翔高新技术产业基地57个项目完成投资41亿元，起步区落地产业项目24个、总投资214亿元，一批企业竣工投产。

　　基础设施更加完善。地铁1号线运行安全有序，2号线实现洞通并完成全部车站主体结构，3、4号线和6号线马銮湾片区段建设进展顺利。翔安机场片区路网建设全面开工。第二西通道进展顺利，第二东通道开工建设，国道324改线等一批重点交通项目投用。建成一批调水项目，提标改造8座污水厂并投入运行，新建改造污水管网96.8公里，清疏排水管网371公里。西部垃圾焚烧发电厂二期建成投用。管道燃气用户数突破50%。新增输电线路20公里，供电可靠率居全国第五。综合管廊总评位居全国第一，海绵城市总评位居全国前列，圆满完成三年试点任务。

　　城市管理水平持续提升。荣获2018中国城市治理智慧化综合奖，市民卡App入围“首届数字中国峰会最佳实践成果30强”。“多规合一”更加完善，厦门特色城市设计体系基本形成，城市“双修”有序推进。第二轮城市改造提升成效明显，实施街区立面改造项目30个，整治主次干道31条、建筑1216栋，提升道路38公里。完成老旧小区改造156个。深入推进市容环境整治，拆除“两违”518.9万平方米。生活垃圾分类位列全国46个重点城市首位，覆盖全市93%的居民小区。深入实施畅通城市三年行动计划，完成36处隐患路段整治和20所学校周边道路交通改善，打通6条断头路，更新投放纯电动公交车884辆，新增调整公交线路127条，新增公共停车泊位1.2万个。

　　乡村振兴加快推进。在建乡村振兴项目547个，总投资375.6亿元。都市现代农业营收829亿元，增长12.1%。7家企业入选全国农业产业化龙头企业500强。获批全国农产品冷链流通标准化示范城市。完成346个自然村分散式污水处理设施和278个自然村截污纳管工程，实现流域内农村生活污水治理全覆盖。30个美丽乡村建设进展顺利，白交祠、马塘入选中国美丽休闲乡村。全面完成农村集体资产清产核资。顺利完成村级组织换届选举。创新乡村治理模式，海沧区入选全国农村社区治理实验区。

　　（五）坚持补短板、惠民生，人民生活水平实现新改善

　　社会事业繁荣发展。建成20个中小学项目，开建20个幼儿园项目。厦门一中海沧校区、科技中学翔安校区正式招生，双十中学、外国语学校、实验小学岛外校区开工建设。建成厦门大学附属翔安医院、弘爱医院、心血管病医院等一批重点医疗项目。新增中小学、幼儿园学位2.75万个，新增医疗床位4000张，均为历年最多。名医工作室柔性引进专家170名，定期为市民开展诊疗服务。顺利通过国家卫生城市复审、国家基本公共卫生服务项目考核。鼓浪屿世界文化遗产得到更加有效的保护利用。市图书馆集美新馆开馆。开建城市健康步道，建成市体育中心综合健身馆。我市运动员获得4个世界冠军、3个亚洲冠军、6个全国冠军。

　　社会保障力度加大。36项为民办实事项目全部完成。城镇新增就业18.7万人，城镇登记失业率控制在年度目标3.8%以内。统一职工、城乡居民大病医保制度，最高赔付限额分别提高到50万元、40万元。城乡居民基本医保筹资标准提高到930元。帮扶困难群众，提高特困人员、低保人员等重点保障对象供养标准和医疗救助比例，社会救助资金支出增长6%。39家农村幸福院和居家社区养老服务照料中心加快建设，新增养老床位1612张。开建保障性住房2.8万套、在建6.25万套、推出1.97万套，是受理量最多、推出数量最多、保障范围最广的一年。

　　社会治理取得实效。我市社区治理创新实践被民政部列入改革开放40周年创新成果。建成全市统一的食品安全信息追溯管理平台，食品安全综合考评和群众满意度均位列全省第一。强化安全生产监管，狠抓重点行业领域专项整治，安全生产事故死亡人数下降18.2%。加强防灾减灾救灾工作，健全应急协调联动机制，完成182个地灾隐患治理项目。扫黑除恶专项斗争成效明显，查处一批涉黑涉恶腐败问题，打掉一批黑恶势力团伙，刑事警情下降23.8%，社会治安持续向好。大力推进诉访分离改革，历史积案有效化解。支持部队改革强军，完成军队全面停止有偿服务工作，国防动员、双拥共建、优抚安置工作进一步加强。民族、宗教、外事、侨务、粮食、人防、气象、档案、地方志工作扎实推进，妇女、儿童、青年、老龄、残疾人、红十字、慈善事业取得新进展。

　　（六）坚持转作风、提效能，政府自身建设呈现新气象

　　持续兴起习近平新时代中国特色社会主义思想“大学习”热潮，扎实推进“两学一做”学习教育常态化制度化。把巡视整改作为“四个意识”的试金石和检验“两个责任”的重要标尺，全面认领中央巡视组反馈的3个方面问题，梳理细化57项整改措施，实行动态管理、挂账销号，集中整改取得阶段性成效，政府系统党的建设进一步加强。激励广大干部勇于担当敢于作为，有力调动干部干事创业积极性主动性，机关效能进一步提升。严格落实中央八项规定精神及省市实施办法，坚决反对形式主义、官僚主义，文风会风显著改善，“三公”经费持续下降。自觉接受人大监督、政协监督、监察监督和社会监督，认真办理人大代表建议333件、政协提案532件，办复率均为100%。提请市人大常委会审议法规草案和法规修正案12件，制定和修改市政府规章5件，废止和宣布失效市政府规范性文件43件，率先全面推行行政执法“三项制度”，政府透明度指数位列全国第一，法治政府建设不断推进。查处侵害群众利益的不正之风和腐败问题55起、处分45人，党风廉政建设和反腐败斗争取得积极成效。

　　各位代表！过去的一年，我们坚持稳中求进工作总基调，稳就业、稳金融、稳外贸、稳外资、稳投资、稳预期，各项工作取得新的成效。成绩来之不易，这是习近平新时代中国特色社会主义思想科学指引的结果，是在市委坚强领导下，在人大代表、政协委员有效监督和大力支持下，全市人民齐心协力、砥砺奋进的结果。在此，我谨代表市人民政府，向全市人民，向全体人大代表和政协委员、各民主党派、工商联、各人民团体、无党派人士、离退休老同志和社会各界人士，向中央和省驻厦单位、驻厦部队、武警官兵，向来厦的投资者和劳动者，向关心支持厦门发展的台港澳同胞、海外侨胞和国际友人，表示衷心的感谢和崇高的敬意！

　　同时，我们也清醒地看到，发展中还存在不少困难和问题，主要是:外部环境复杂多变，地区生产总值、固定资产投资等指标未能达到年初预期，经济快速增长基础不够稳固；转方式、调结构、促创新任务仍然比较繁重，城乡、区域发展不平衡不充分问题仍然存在；改革开放力度仍需加大，经济特区引领带动作用还需增强；群众在就业、养老、教育、医疗、居住等方面仍然面临不少难题，基本公共服务均等化需要进一步推进；国有企业重点领域和关键岗位的监管机制需进一步强化；部分干部改革创新、干事创业的精气神不足，隐形变异“四风”问题还时有发生。我们要正视这些问题，认真加以解决。

　　二、2019年主要工作

　　今年是新中国成立70周年，是决胜全面建成小康社会、坚持高质量发展落实赶超的关键之年。做好今年工作，要坚持以习近平新时代中国特色社会主义思想为指导，深入学习贯彻党的十九大和十九届二中、三中全会精神，全面贯彻落实习近平总书记对福建、厦门工作的重要指示精神，加强党对经济工作的集中统一领导，统筹推进“五位一体”总体布局，协调推进“四个全面”战略布局，坚持稳中求进工作总基调，坚持新发展理念，坚持高质量发展落实赶超，坚持以供给侧结构性改革为主线，坚持深化市场化改革、扩大高水平开放，加快建设现代化经济体系，继续打好三大攻坚战，抓实“双千亿”工作，积极牵头推进闽西南协同发展区建设，进一步稳就业、稳金融、稳外贸、稳外资、稳投资、稳预期，统筹推进稳增长、促改革、调结构、惠民生、防风险、保稳定工作，提振市场信心，不断增强人民群众获得感幸福感安全感，保持经济持续健康发展和社会大局稳定，努力建设高素质高颜值现代化国际化城市，以优异成绩迎接中华人民共和国成立70周年。

　　综合各方面因素，今年发展的主要预期目标为:地区生产总值增长8%左右，固定资产投资增长10%左右，财政总收入和地方级财政收入分别增长5%和3.5%左右，社会消费品零售总额增长10%，城镇居民和农村居民人均可支配收入均增长8%，居民消费价格涨幅控制在3%左右，完成国家和省下达的节能减排任务。

　　重点做好八个方面工作:

　　（一）高质量发展实体经济，着力构建现代化经济体系

　　始终把实体经济作为高质量发展落实赶超的第一支撑，把创新作为第一动力，把产业作为第一战场，持续提升效益链价值链。一是大力实施创新驱动战略。以自主创新示范区建设为抓手，集聚创新资源，活跃创新要素，推动第二批20项创新事项落地见效，策划生成第三批事项，加快建设区域科技创新中心。强化企业创新主体地位，激励企业加大研发投入，加强技术储备；培育一批“独角兽”“单项冠军”“专精特新小巨人”企业，新增200家国家高新技术企业。构建开放、协同、高效的共性技术研发平台，健全产学研一体化创新机制，加快培育引进新型高水平研发机构，推动中国航天十二院厦门分院、沈阳新松机器人产业研究院等项目落地，新增10家国家级研发平台。优化创新创业生态，加快发展创新工场、加速器等新型孵化模式，新增30家专业化众创空间、10家科技企业孵化器；推动创新链、产业链、资金链、政策链有机融合，促进高校、科研院所科技成果在厦转化；加强知识产权创造、保护与运用，深化质量提升和品牌创建，大力推进开放创新，激发全社会创新创业活力。二是全力推进产业发展高端化。要把制造业高质量发展放到更加突出位置，推动先进制造业与现代服务业深度融合。要增强主导产业竞争力，加快平板显示产业技术创新，推动计算机与通讯设备产业软硬件融合，重点布局新型显示技术，大力发展高端服务器与存储设备等核心产业，提升市场竞争力；推动机械装备产业突破一批核心基础零部件和先进基础工艺，加快实现智能制造。要实施新兴产业倍增工程，提升半导体和集成电路产业规模和水平，推进产业链各环节协同发展；加快引进一批医药龙头企业，大力发展仿制药，支持研发创新药；积极对接国家新材料产业“折子工程”，力争在高端稀土、锂离子电池材料等领域取得新突破；大力发展动漫游戏、设计服务、影视演艺、艺术品等文化创意产业；积极培育软件信息平台型企业，支持软件企业向大数据、移动互联网、物联网、云计算等领域发展，促进人工智能与经济社会发展深度融合，争创国家数字经济发展示范区。要促进现代服务业集聚发展，培育引进大型高端学术会议和展览项目，积极创建全域旅游示范市；推动物流业线上线下融合，加快建设东南区域物流枢纽中心；着力构建多层次资本市场体系，加快建设两岸区域性金融服务中心，提升金融服务实体经济水平。三是全力帮扶企业。深入开展企业服务年活动，推出重点行业和企业精准帮扶措施，健全分类帮扶工作机制，协调解决生产经营中存在的困难问题；帮助企业减轻负担，落实降本减负政策，及时兑现已出台的各项惠企措施；帮助企业转型升级，加快实施新一轮技改专项行动，鼓励企业增资扩产，助推一批优质科技企业到科创板上市；帮助企业融资对接，深化政银企合作，建立联合授信机制，用好应急转贷资金、政策性融资担保和纾困基金等，为企业发展提供更充裕资金保障；帮助企业开拓市场，发布地产工业品目录，鼓励产业链上下游互采互用，支持企业参加境内外各类展会；帮助企业招工引才，大力实施30万院校毕业生招聘行动，加强人才需求开发和对接，培育壮大面向生产一线的实用工程人才和专业技能人才队伍，让多层次人才成就未来。要支持民间资本进入教育、医疗、养老等领域，鼓励民间资本参与混合所有制改革。构建亲清新型政商关系，出台更多有温度、能见效的政策措施，让各种所有制经济依法平等使用生产要素、公平参与市场竞争、同等受到法律保护，充分激发企业、企业家活力和创造力。

　　（二）狠抓投资消费出口，推动经济持续健康发展

　　推动供给与需求有效对接、投资与消费有机结合、内需与外需协调拉动，筑牢经济持续稳定增长基础。一是持续抓好“双千亿”工作。深化项目带动，更好发挥投资关键作用，更加注重拉动有效投资，提升投资规模质量效益。针对产业链缺失和关键环节，精准强链补链扩链，力促工业投资增长10%。狠抓招商引资，注重以商招商、二次招商与产业链招商，瞄准世界500强、全国百强，引进一批投资强度大、科技含量高、创新能力强的项目。狠抓“五个一批”项目攻坚，推动翔安机场主体工程、“一场两馆、新会展中心”、黄厝国际会议中心等一批重大基础设施项目，厦钨新能源材料产业园、金鹭硬质合金产业园等一批重大产业项目，滨海旅游浪漫线三期、同安一中滨海校区等一批民生补短板项目集中开工建设。加快存量低效用地转型，支持存量土地二次开发，依法依规收回逾期未用土地，着力提高土地和投资产出效率。加强对114个在谈项目的跟踪促进，计划往前排、工期往前赶、服务往前冲，强化要素保障，推动项目早签约、早开工、早投产。二是积极推动消费升级。加快消费领域基础设施建设，优化城市核心商圈功能，完善岛外四区商业设施配套，加快农贸市场改造提升，推动商贸转型升级。大力培育中高端消费，推动邮轮中心保税综合体等一批优质项目落地。放宽服务消费市场准入，大力发展文化、教育、信息、健康等新兴消费。完善“互联网+”消费生态体系，促进电商、快递进社区进农村，推动线上线下销售融合发展。三是推动出口稳定增长。积极有效应对外部经济环境变化，用足用好出口信保、开拓市场等扶持政策，优化退税、通关、结汇等经营环境，大力拓展出口订单和异地货源。调整出口商品结构，扩大具有自主知识产权、自主品牌和高附加值产品出口。支持外贸综合服务、设备融资租赁、服务外包、国际中转集拼等新型贸易业态发展。坚持进口出口并重，削减进口环节制度性成本，鼓励大宗商品和消费品进口，扩大先进技术设备、关键零部件进口，推动建筑设计、商贸物流、咨询服务、研发设计、环境服务等生产性服务进口，促进对外贸易平衡发展。

　　（三）落实经济特区新定位，进一步深化改革扩大开放

　　认真贯彻落实习近平总书记在庆祝改革开放40周年大会上的重要讲话精神，按照经济特区成为“改革开放的重要窗口、试验平台、开拓者、实干家”的战略定位，持续深化市场化改革，扩大高水平开放。一是推进重点领域和关键环节改革。深化供给侧结构性改革，按照中央八字方针要求，持续巩固“三去一降一补”成果、增强微观主体活力、提升产业链水平、畅通经济循环。加快推进中央赋予的重大改革任务，推动经济体制、对外开放体制等11个重点领域改革，着力增强改革系统性、整体性、协同性。深化财税体制改革，加强财政专项资金整合，全面实施预算绩效管理，做到增收节支提效。深化国资国企改革，完善国企法人治理结构，推进混合所有制改革，抓好国有资本投资运营试点，强化经营风险管控和惩处违法违规行为，提高国有资本配置效率和国企竞争力。深化医药卫生体制改革，做好药品集中采购试点工作，加快紧密型医联体建设，完善公立医院管理体制和薪酬制度。建设信用大数据创新中心，加快信用数据政务共享和市场化应用。加快国家生态文明试验区建设，持续深化生态文明体制改革。二是打造国际一流营商环境。认真对照国家营商环境评价体系，精准补齐短板弱项，确保全国领先。深入实施准入前国民待遇加负面清单管理制度，持续放宽市场准入，保护外资企业合法权益，依法惩处侵犯知识产权行为。持续深化“放管服”改革，坚持能减则减、能简则简、能免则免、能并则并，深入推进“多证合一” “证照分离”改革，推动工程建设项目审批制度改革继续走在全国前列。深化“双随机一公开”监管改革，推进跨区域跨部门联合执法。加快审批服务便民化改革，大力推广电子证照，推动更多服务事项“马上办、网上办、就近办、一次办”，实现更多事项“一趟不用跑、最多跑一趟”。三是把自贸试验区建设成为新时代改革开放新高地。强化“三区联动”，加大改革措施系统集成，力争在投资、贸易、金融、事中事后监管等领域推出更多创新举措，提升投资贸易自由化便利化水平。更好发挥“保税+” “金融+”等综合优势，推动航空维修、跨境电商、融资租赁、机电设备等重点平台建设。充分发挥厦门中欧班列辐射带动作用，大力发展海铁联运，打造“海丝”与“陆丝”无缝对接的国际物流大通道。深入推进口岸通关提效降费，建设“单一窗口”3.0版。四是积极参与“一带一路”建设。围绕打造“海丝”战略支点城市，加快建设互联互通、经贸合作、海洋合作、人文交流四个枢纽。加快国际航运中心建设，增加“海丝”航班，打响“丝路海运”品牌。加强与沿线国家的产能合作和创新交流，办好厦洽会，大力促进双向贸易投资。加快建设中国-东盟海洋合作中心，强化海洋生态环境保护和产业合作。发挥侨务优势，深化与沿线国家科技、教育、文化、卫生、旅游等领域合作，促进民心相通。五是推进闽西南协同发展区建设。强化项目对接、信息沟通、事务协调，在更高站位上推动区域一体化发展。健全产业园区共建和利益共享机制，落实各项对口合作协议，加快城际轨道R1线建设，深化九龙江水源保护、海域污染联防联控等合作，扎实推进一批重大项目建设，推动五市全方位对接协作，提高区域整体竞争力。

　　（四）发挥前沿平台优势，持续深化对台交流合作

　　全面贯彻中央对台大政方针，大力促进经贸合作畅通、基础设施联通、能源资源互通、行业标准共通，为推动两岸关系和平发展作出更大贡献。一是增进经贸合作。持续推进两岸交流合作综合配套改革，加快“一区三中心”建设，深化先进制造业和现代服务业等方面合作，打造两岸共同市场。落实2018年两岸企业家峰会年会投资意向，精准引进一批台湾百大项目。拓展台湾入境商品“源头管理、口岸验放”模式，扩大台湾进口商品第三方检验检测结果采信范围，建设两岸货物集散转运枢纽。二是推动厦台融合。抓好“台商台胞服务年”活动，为台胞台企提供同等待遇。办好第十一届海峡论坛、文博会、海图会等重要活动。推动厦台教育文化、医疗卫生合作，社会保障和公共资源共享，力争率先与金门等地实现基本公共服务均等化、普惠化、便捷化。投用五通客运码头三期，全力推动厦金通电、通气、通桥，探索建立厦金旅游协作示范区。实施台青实习“银鹰计划”、赴台征才千人计划，继续打造一批国家级两岸青年就业创业基地和对台研学旅行基地，力争每年吸引5000名青年来厦实习、就业、创业。继续推动厦台社区村里结对共建，促进两岸同胞心灵契合。

　　（五）全力推进跨岛发展，提升城市发展科学化精细化智能化水平

　　围绕争创一流，统筹城市规划建设管理，着力提升城市能级，增强城市辐射力带动力。一是全面加快新城基地建设。集美新城要完成投资235亿元，加快电子城等166个项目建设，抓好软件园三期等片区产业集聚，完善生产生活综合配套。环东海域新城要完成投资535亿元，加快泰康之家养老等311个项目建设。马銮湾新城要完成投资280亿元，加快生态修复二期工程等223个项目建设。同翔高新技术产业基地要完成投资60亿元，加快石墨烯产业园等63个项目建设。二是完善现代化基础设施。开通运营地铁2号线，实现3号线全线洞通，加快建设4号线和6号线。加快建设第二东、西通道，推进第三东通道前期工作，加快福厦高铁客运专线建设进度。完成枋洋水利枢纽主体工程，建成西水东调一期工程，推进九龙江北溪雨洪利用工程、进岛输水第二通道等前期工作，加快实施提升城市供水水质三年行动方案。新建管道燃气60公里，新增用户3万户。开工建设500千伏集美输变电工程，提升配电网安全供电能力。开工建设15公里综合管廊。三是加快建设智慧城市。加快推进5G技术商用，加快建设大数据中心和先进计算中心，促进技术融合、业务融合、数据融合。大力发展智慧民生，在城市规划管理、医疗卫生、社会治安、交通出行、政务服务等领域打造一批应用示范项目。四是提升城市管理水平。强化沿海沿湖沿街等重要节点规划设计，统筹推进城市地上地下设施综合改造提升，完成铁路沿线环境综合整治。推进旧城旧村和老旧小区更新改造，优化物业管理。提升城市园林绿化水平，创建国家生态园林城市。深入推进“门前三包”，加强占道经营、户外广告、油烟噪音扰民等专项整治，岛内杜绝流动摊贩，鼓浪屿杜绝露天烧烤。严厉打击“两违”，发现一起、查处一起、问责一起，确保“两违”零增长。加快进出岛公交换乘枢纽建设，实施仙岳路、成功大道出入口、进出岛桥隧等堵点改善工程，整治30处交通安全隐患路段，加强出租车、网约车等行业管理。

　　（六）全力实施乡村振兴战略，加快推进农业农村现代化

　　按照产业兴旺、生态宜居、乡风文明、治理有效、生活富裕的总要求，深入实施12个三年行动计划，推动农业全面升级、农村全面进步、农民全面发展。一是大力发展都市现代农业。深入推进农业绿色化、特色化、品牌化，培育10个“三品一标”认证产品，加快实施25个农业产业化项目，大力发展农产品精深加工和综合利用，为市民提供更多优质农产品。

　　（上接A02版）二是加快建设美丽宜居乡村。抓好8个市级重点示范村、12个市级示范村建设，完善村庄规划、建设和管护机制。抓好农村人居环境整治“一革命四行动”，新建改造一批农村公厕，推行农村垃圾城乡一体化处理，推进“三边”整治和“四旁”绿化，全面提升农村人居环境质量。三是推动城乡融合发展。加快基础设施向乡村延伸、资源要素向乡村流动，持续完善农村供水供电、道路通讯等基础设施和教育医疗、健康养老等公共服务设施。鼓励引导社会资本、技术、人才参与农村建设，实现省级科技特派员覆盖全部镇街。深化农村集体产权制度改革，落实农村土地“三权”分置制度，培育新型职业农民和新型经营主体，推动农村劳动力就业转移，多渠道促进农民增收。发展优秀乡村文化，创新乡村治理，深入开展移风易俗行动。

　　（七）切实保障和改善民生，推动社会治理共建共治共享

　　始终坚持以人民为中心的发展思想，加快补齐民生短板，着力解决好事关百姓生产生活的关键问题，让市民有更多获得感幸福感安全感。一是加快推进教育现代化。落实立德树人根本任务，深化教育改革创新，构建德智体美劳全面培养的教育体系。建成16个中小学项目、开工建设18所幼儿园，新增学位2.8万个。大力实施名校跨岛战略，加快双十中学翔安校区、实验小学翔安校区、外国语学校集美校区建设。持续实施小学幼儿园课后延时服务。规范各类校外培训，为家长分忧、为学生减负。树立健康第一的教育理念，增强学生体质、健全学生人格。支持在厦高校加快“双一流”建设和应用型本科高校建设。提高职业教育办学质量，深化产教融合，加快培养高素质劳动者和高技能人才。弘扬尊师重教的社会风尚，努力提高教师政治地位、社会地位、职业地位，确保中小学教师平均工资收入水平不低于公务员。二是持续推进健康厦门建设。支持第一医院、中山医院等本地核心医院发展，加快马銮湾医院、环东海域医院等在建项目进度，开工建设市妇幼保健院集美院区和一批社区卫生服务中心。扩大复旦中山厦门医院、北京中医药大学厦门医院、名医工作室的知晓度和影响力，让更多市民享受到名医诊疗服务。支持社会资本举办中医、精神卫生、康复护理等短缺专科医疗机构。健全疾病防控和卫生应急体系，提高基本公共卫生服务水平。三是推动文体事业繁荣发展。永续传承鼓浪屿历史文脉，强化历史文物保护和活化利用，让琴声、书声、歌声代替叫卖声。完善现代公共文化服务体系，广泛开展各类群众性文化活动，办好金鸡百花电影节，倡导全民阅读，建设书香城市，创作一批文艺精品，加快闽南戏曲艺术中心建设。大力开展全民健身运动，继续推动学校体育设施向社会开放。办好第20届市运会。四是提高社会保障水平。健全就业服务体系，强化就业和创业技能培训，精准帮扶高校毕业生、被征地农民、海域退养渔民、失业人员等重点群体就业创业，建立劳动报酬正常增长机制，构建和谐劳动关系。完善医保筹资和支付制度，推进异地就医直接结算。提高低保对象和特困人员救助供养标准，加强残疾人康复服务。加快扩建厦门老年大学，继续推进居家社区养老服务照料中心、农村幸福院、高端养老项目建设，支持有条件的小区进行适老化改造，新增养老床位1300张。加快推进6.25万套在建保障性住房建设，新建2.1万套，推出更多房源配租配售。落实国家粮食安全新战略，保障军需民食。加强食品药品安全监管，建成国家食品安全示范城市。五是全面加强和创新社会治理。坚持重心下移、力量下沉，完善社区治理体系，打通服务市民的“最后一公里”。健全应急管理体制机制，完善防灾减灾救灾体系，提高应急救援能力，守护人民群众生命财产安全。深入推进扫黑除恶专项斗争，大力惩治各种违法犯罪活动，加快构建立体化、智能化社会治安防控体系。完善预防和化解社会矛盾机制，创新信访工作方式，保障人民群众合法利益。更好发挥工青妇等群团组织和民族宗教在社会治理中的作用，持续创建全国文明城市。加强双拥共建，支持驻厦部队练兵备战，做好退役军人事务，加快建设军民融合发展示范市。

　　（八）坚决打好三大攻坚战，为全面建成小康社会提供有力保障

　　以更大的力度、更实的措施，确保风险隐患得到有效控制，确保脱贫攻坚任务全面完成，确保生态环境质量持续提升。一是加强重大风险防范化解。加大不良贷款化解力度，加快地方金融机构非现场监管系统建设，规范发展各类交易场所，深化互联网金融专项整治，严厉打击非法集资等违法活动。加强地方政府债务管理，规范举债程序和资金用途，提升政府债务资金使用效益。坚持“房住不炒”定位，围绕稳地价、稳房价、稳预期目标，建立房地产管理长效机制，培育和发展住房租赁市场，促进房地产市场平稳健康发展。二是坚决扛起对口扶贫协作政治责任。做好援宁援藏援疆援渝各项工作，聚焦甘肃临夏州建档立卡贫困人口精准发力，多措并举促进对口协作地区贫困人口稳步脱贫。落实好省内扶贫开发工作任务，同时要高度重视本市困难群众帮扶，确保“两不愁、三保障”。三是推进污染防治取得更大成效。持续抓好中央和省环保督察反馈问题整改。坚决打赢蓝天保卫战，实施新一轮大气污染防治行动，加强工业、机动车、扬尘“三大污染源”治理，建设绿色海港空港，确保空气质量保持全国前列。着力打好碧水保卫战，实行“双总河（湖）长”制，深入实施水污染防治行动计划，加快饮用水水源地保护区“划、立、治”工作，持续深化小流域综合治理，推进前埔、西柯等污水厂新改扩建工程，新建改造城区污水管网90公里。加强陆海统筹，实施海域清淤整治，加强湿地、沙滩、海湾、海岛生态保育。扎实推进净土保卫战，稳步开展土壤污染状况详查、风险防控和治理修复。严禁新批矿产资源勘探开采，加强山体保护。建成投用东部垃圾焚烧厂二期，加快建设工业废物、医废处置二期项目，实现生活垃圾分类覆盖率100%、原生垃圾“零填埋”。

　　三、全面建设新时代人民满意政府

　　始终坚持以习近平新时代中国特色社会主义思想为指导，坚决把高质量发展落实赶超的重任扛在肩上，把解决群众关心关注的热点问题抓在手上，全面加强政府自身建设，切实做到信念过硬、政治过硬、责任过硬、能力过硬、作风过硬，推动厦门发展再上新台阶。

　　加强政治建设。牢固树立“四个意识”，坚定“四个自信”，坚决做到“两个维护”，始终在政治立场、政治方向、政治原则、政治道路上同以习近平同志为核心的党中央保持高度一致，全力推动党中央国务院、省委省政府和市委各项决策部署落地见效。坚决按照全面从严治党“八个坚定不移”的要求，落实主体责任，加强政府系统党的建设，严格遵守政治纪律和政治规矩，严格执行《准则》《条例》，严格落实意识形态工作责任制。狠抓中央巡视反馈意见后续整改，坚决防止问题反弹回潮。

　　坚持依法行政。严格遵守宪法法律，深化全民普法，加强重点领域立法，确保政府各项工作在法治轨道上运行。自觉接受人大监督、政协监督和监察监督，落实重大决策出台前向市人大及其常委会报告制度，办好人大代表议案、建议和政协提案，主动接受民主党派、人民群众和社会舆论监督。进一步强化审计监督，用好审计成果。坚持科学、民主、依法决策，对涉及公众利益的重大事项，广泛听取各方面意见。加大政务公开力度，严格规范公正文明执法，持续提升政府公信力。

　　增强能力本领。面对不断出现的各种困难、风险和挑战，必须持续增强学习本领、政治领导本领、改革创新本领、科学发展本领、依法执政本领、群众工作本领、狠抓落实本领、驾驭风险本领。要坚持问题导向，强化目标导向，更加注重精准施策，更加注重统筹协调，牢牢把握工作主动权，做到善谋善为、善作善成。

　　强化担当作为。大力弘扬“马上就办、真抓实干”优良作风，狠抓效能建设，深入开展“加大晒的力度、加快赶的进度”活动，定期晒指标进度、晒项目开竣工，以此晒出问题差距、晒出思路对策、晒出落地项目，赶出闯劲、赶出冲劲、赶出质量效益。要大力提振干部精气神，激励干部解放思想、开拓进取，在“比学赶超”中狠抓落实。要坚持严管和厚爱相结合，完善激励机制和容错纠错机制，让敢担当有作为的干部干得安心、拼得开心，让敢担当有作为的干部有干劲、有奔头。

　　建设廉洁政府。认真贯彻落实中央八项规定精神及省市实施办法，集中整治形式主义、官僚主义，严防“四风”问题回潮反复。保持无禁区、全覆盖、零容忍高压态势，严防关键岗位和重点领域廉政风险，严查各类违规违纪行为，严惩群众身边腐败问题。认真履行“一岗双责”，用好监督执纪“四种形态”，强化廉政警示教育，让广大公职人员知敬畏、存戒惧、守底线，形成风清气正的良好政治生态。

　　各位代表，新思想指引前进方向，新时代勇挑责任重担。让我们紧密团结在以习近平同志为核心的党中央周围，高举习近平新时代中国特色社会主义思想伟大旗帜，在省委省政府和市委的坚强领导下，苦干实干、敢闯敢拼，锐意进取、奋力赶超，加快建设高素质高颜值现代化国际化城市，勇当新时代中国特色社会主义排头兵！
